# Climate change boredom: Exploring its predictors and the psychological factors that influence intention to act

**DOI:** 10.1371/journal.pone.0348574

**Published:** 2026-05-27

**Authors:** Ruxandra Malina Petrescu-Mag, Dacinia Crina Petrescu, Hamid Rastegari, Adrian Ivan, Ioan Valentin Petrescu-Mag

**Affiliations:** 1 Department of Environmental Science, Faculty of Environmental Science and Engineering, Babes-Bolyai University, Cluj-Napoca, Romania; 2 Gembloux Agro-Bio Tech, University of Liège, Gembloux, Belgium; 3 Doctoral School “International Relations and Security Studies”, Babes-Bolyai University, Cluj-Napoca, Romania; 4 Department of Hospitality Services, Faculty of Business, Babes-Bolyai University, Cluj-Napoca, Romania; 5 Faculty of Geographical Sciences and Planning, University of Isfahan, Isfahan, Iran; 6 Department of International Studies and Contemporary History, Faculty of History and Philosophy, Babes-Bolyai University, Cluj-Napoca, Romania; 7 Department of Environmental Engineering and Protection, Faculty of Agriculture, University of Agricultural Sciences and Veterinary Medicine Cluj-Napoca, Cluj-Napoca, Romania; 8 Doctoral School of Engineering, University of Oradea, Oradea, Romania; West University of Timisoara: Universitatea de Vest din Timisoara, ROMANIA

## Abstract

Multiple psychological factors, from fear to hope, play a role in actions addressing climate change. However, an understanding of how these factors interact to shape such behaviors remains limited. This gap holds significant implications for advancing both research and climate practice. The present study aims to contribute to the overall effectiveness of climate change mitigation and adaptation efforts by providing an understanding of the factors influencing individuals’ intentions to act against climate change, and casting light upon a less explored dimension – climate change boredom. Climate change boredom highlights a psychological barrier that can hinder climate action. Intention to act is a key step toward a change in behavior and effective environmental efforts. Therefore, we determined the factors that predicted climate change boredom and investigated the influence of climate change boredom on people’s intention to act against climate change. Using Partial Least Squares Structural Equation Modeling (PLS-SEM) on data obtained from a survey of a representative sample of Romanian people, the study found that beliefs about climate change, climate change goal commitment, self-efficacy of cooperation, perceived health impacts of climate change, living environment, and age predicted 66.8% of climate change boredom. Results showed that climate change boredom significantly hindered the intention to act against climate change. From a practical perspective, identifying predictors of climate change boredom and intention to act is important in developing strategies, policies, and communication approaches that reinvigorate individuals’ and communities’ motivations to participate in climate action efforts. Moreover, addressing climate change boredom is essential for ensuring long-term environmental security, as disengagement from climate issues can weaken societal resilience and hinder adaptive strategies in the face of climate threats.

## 1. Introduction

Climate change, one of our time’s most pressing global threats, evokes a wide range of emotions, from anxiety, anger, irritation, and boredom, to hope [1–4], reflecting its extensive environmental, social, and economic implications [5–7]. Understanding how these emotional responses translate into climate-related behaviors has become a central challenge in environmental psychology. In light of the diverse emotional responses elicited by climate change, recent studies have sought to systematically map and experimentally elicit these emotions across populations. Thus, Baum et al. [8] mapped five emotions (fear, hope, anger, sadness, worry) across 30 countries with 30,284 participants, providing the first global-scale evidence. Zaremba et al. [9] developed validated stimuli evoking five emotions (anger, anxiety, compassion, guilt, hope) across three studies. Recent research has further demonstrated that specific emotional profiles are associated with support for climate policies and environmental decision-making, highlighting the functional role of emotions in shaping public responses to climate change.

Studies have demonstrated the role of various psychological factors in sustainable behavior, including climate action. Limited cognition, ideological beliefs that discourage pro-environmental attitudes and actions, and skepticism toward experts and authorities hinder climate change mitigation and adaptation [10]. Building on these insights, recent empirical research has further explored how targeted psychological interventions can influence climate-related beliefs and behaviors. Cutler et al. [11] tested psychological interventions with 3,055 participants across six countries and found that belief in climate change and support for pro-environmental policies were associated with increased climate motivation. These findings underscore the importance of psychological processes in bridging the gap between climate awareness and action. Factors such as fragmented, contradictory, or insufficient knowledge about the likely impacts of climate change or the best ways to avoid them can lead to cognitive dissonance [12] and inconsistency between beliefs and behaviors [13].

Cognitive dissonance is an unpleasant psychological state generated by an inconsistency between two or more elements of one’s cognitive system (e.g., beliefs) [14,15]. This dissonance prompts people to act to reduce it, for example, by avoiding or altering the perceived importance of certain information [[16] citing [15]]. For instance, people may recognize that climate change is largely driven by human activities while continuing to engage in high-carbon behaviors such as frequent car use or consumption of unsustainable products. To reduce this inconsistency, they may downplay the impact of their personal actions or justify them on the grounds of convenience or a perceived lack of alternatives [12], a pattern recently discussed in studies on the environmental value-action gap and climate-related cognitive dissonance. Such dissonance-reduction strategies can ultimately lead individuals to maintain or even reinforce environmentally harmful behaviors, including continued engagement in carbon-intensive practices (e.g., excessive car use, frequent flying, or overconsumption) despite awareness of their negative environmental impact [16]. In addition to skepticism [17] and cognitive dissonance, actions to reduce climate change can also be hindered by boredom. However, compared to other emotional and cognitive barriers, boredom remains relatively underexplored in the climate change literature.

Boredom was defined as “an affective indicator of unsuccessful attentional engagement in valued goal-congruent activity” [18]. Boredom is an unpleasant emotion expressed by a desire to block out or ignore what is going on and a lack of complete engagement in a subject [[19] citing [20,21]]. Thus, boredom may indicate disengagement from climate change [19]. Practically, in the context of climate change, boredom may arise from several interrelated mechanisms. Repeated exposure to similar climate messages in media and public discourse can lead to information fatigue, reducing attentional engagement and perceived novelty. At the same time, the abstract, long-term, and often overwhelming nature of climate change may diminish perceived personal relevance and efficacy, prompting individuals to disengage to regulate emotional discomfort, such as anxiety or helplessness. Research suggests that such disengagement can function as an emotional coping strategy, whereby individuals distance themselves from climate information to avoid negative affect or cognitive overload [22,23]. Geiger et al. [24] developed one of the first studies that measured and compared emotional reactions and whether they predicted intention to act. They demonstrated that stronger feelings of boredom predicted decreased intention to act. Despite this initial evidence, there is still limited understanding of what drives climate change–related boredom and how it interacts with other psychological factors to influence behavior.

The intention to act is often accepted as the best predictor of behavior [25] (in this case, actions to reduce climate change). The intention shows that a person is determined and committed to taking steps that reduce the impacts of climate change. Similar to Geiger et al. [24], the present study assesses behavioral intentions (rather than self-reported past behavior) to examine how current feelings or perceptions might affect future behavior. Previous research on emotional reactions (e.g., anxiety, hope) reported mixed results in predicting climate change actions [26–28]. In light of these inconsistencies, recent research has shifted attention toward additional variables, such as the distinct role of hope as a potentially more robust predictor of climate-related behaviors. For instance, Garfin et al. [29] reported hope associated with both individual and collective sustainability behaviors across 1,479 participants. Other studies demonstrated that action-focused hope predicts civic engagement [30] and that hope is a stronger predictor of policy support than anxiety [31]. The apparent inconsistency reflects differences in emotion specificity (general *vs.* action-focused hope), outcome types (individual *vs.* collective behaviors), and emotion regulation strategies rather than fundamental contradictions. Armstrong et al. [32] found emotion regulation strategies mediate these relationships, suggesting context matters substantially. These findings suggest that emotional influences on climate action are complex and context-dependent, reinforcing the need to examine less-studied emotions such as boredom within a broader psychological framework.

Behavioral intention is widely regarded as a proximal determinant of behavior; however, it does not fully translate into actual action. According to the Theory of Planned Behavior, intention is shaped by attitudes, subjective norms, and perceived behavioral control, and represents the most immediate predictor of behavior [25]. At the same time, a growing body of research highlights an intention–behavior gap due to psychological, situational, and contextual constraints, particularly in the context of pro-environmental and climate-related actions [33–35]. Similarly, studies applying TPB in environmental contexts emphasize that additional variables – such as social context, guilt, knowledge about the environmentally friendly behavior, climate change literacy, and institutional support – play a critical role in translating intention into actual behavior [36–38]. Recent applications of TPB in environmental and tourism contexts further confirm its usefulness in explaining pro-environmental intentions, while also highlighting the role of extended factors such as values and experience in shaping behavior [39]. Therefore, behavioral intention should be understood as a necessary but not sufficient predictor of behavior. In line with prior research (e.g., [24]), the present study focuses on behavioral intention as an appropriate outcome variable for examining how cognitive and emotional factors relate to climate change engagement, while explicitly acknowledging the limitations of the intention–behavior gap.

To contextualize the present study, it is important to note that climate change perceptions and behaviors in Romania are shaped by a combination of awareness, concern, and structural constraints. Previous research shows that while many Romanians acknowledge climate change and express concern about its impacts, this does not consistently translate into proactive mitigation or adaptation behaviors, reflecting a persistent value-action gap [40,41]. Evidence from studies on Romanian farmers highlights similar patterns, showing that climate change beliefs and adaptation behaviors vary considerably depending on knowledge, perceived risks, and access to resources [42,43]. Studies on farmers further highlight heterogeneous adaptation patterns, ranging from proactive strategies to passive or delayed responses, influenced by perceived risks, resources, and institutional support [44]. At the same time, generational differences indicate that younger cohorts (Millennials and Gen Z) tend to express higher levels of concern, particularly regarding health implications, but this concern is not always accompanied by sustained engagement [45]. Taken together, these findings suggest that climate change engagement in Romania is characterized by moderate awareness but uneven behavioral responses, which may increase the relevance of psychological barriers, such as boredom, and limit the direct transferability of the findings to contexts with stronger pro-environmental norms.

The present study aims to provide an understanding of the dynamics that shape individuals’ intentions to act against climate change, shedding light on a relatively unexplored dimension, namely, climate change boredom. Specifically, this study addresses a critical gap in the literature by systematically investigating both the antecedents of climate change boredom and its impact on behavioral intentions. This exploration can provide a foundation for designing targeted interventions, communication strategies, and policies that address climate change boredom and reinvigorate individuals’ intention to act, thus improving the overall effectiveness of climate change mitigation and adaptation efforts. To respond to this aim, *two objectives were set. The first objective is to determine the factors that predict climate change boredom, and the second objective is to investigate the influence of climate change boredom on individuals’ intentions to act against climate change.*

## 2. Theoretical background and hypothesis development

This study is grounded in an integrative theoretical framework that combines insights from environmental psychology, social identity theory, and cognitive-affective models of behavior to explain how emotional, cognitive, and social factors jointly shape individuals’ intentions to act against climate change. Specifically, it was assumed that pro-environmental behavior is influenced by the interplay among identity-based motivations, emotional responses (e.g., worry, boredom), cognitive beliefs, and perceived efficacy, all embedded within a broader social context.

Within this framework, environmental identity is conceptualized as a key antecedent of emotional engagement, while climate change worry and boredom represent distinct affective pathways that can either facilitate or hinder action. At the same time, cognitive factors (e.g., beliefs, goal commitment) and social-contextual variables (e.g., sense of community, self-efficacy of cooperation) are expected to reinforce or attenuate these emotional processes. By integrating these dimensions, the present study responds to recent calls for more comprehensive models that move beyond single predictors of climate action and instead capture the complexity of psychological drivers of engagement.

**Fig 1:**
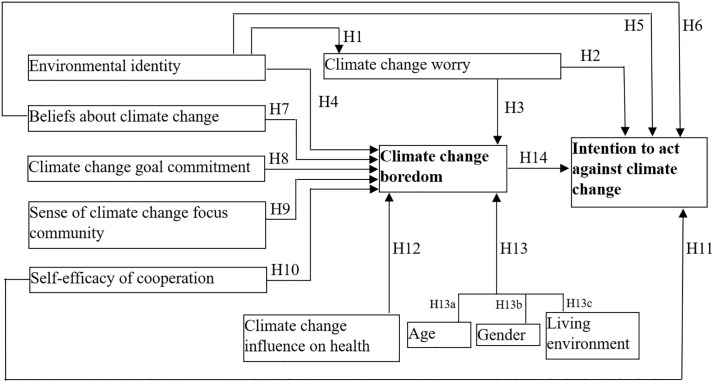
Hypotheses of the study.

We hypothesized the significant positive role of environmental identity in shaping individuals’ emotional responses to climate change (more precisely, climate change worry); the negative association between environmental identity, climate change worry, beliefs about climate change, climate change goal commitment, self-efficacy of cooperation, sense of climate-focused community, climate change influence on health, personal characteristics (age, gender, living environment) and climate change boredom; the relationship between climate change worry, environmental identity, beliefs about climate change, self-efficacy of cooperation, climate change boredom, and people’s propensity to take actions against climate change (Fig 1). Although prior studies have examined some of these relationships in isolation, their combined effects, and particularly the role of climate change boredom within this network, remain insufficiently explored. These components have not yet been fully explored in the context of climate change, nor have their connection been investigated.

Environmental identity refers to the extent to which an individual embeds environmental concerns into their sense of self, which can influence any action that a person considers as influencing or being important to the environment [46]. Prior research found that people who are more connected to nature are more likely to be aware of and affected by dangers to their environments [47–49]. Identifying as an environmentalist was found to be closely linked to pro-environmental behavior, either directly or indirectly through beliefs about global warming [50]. Thus, we assumed that when people feel a strong connection to the environment and consider it a part of their identity, they are more likely to view climate change as a threat to something they deeply care about. This emotional connection can lead to increased worry and concern. In the context of climate change, according to Bouman et al. [51], worry implies that a person is actively and emotionally engaged with the topic of climate change and is personally troubled by its consequences, making this individual appear motivated to act on the issue. Ojala et al. [52] understand worry as repetitive thinking about uncertain future (climate change) adverse events. According to Clayton and Karazsia [53], worry about climate change is a fairly common experience [a detailed analysis of the concepts of worry, anxiety, and grief is presented by [52]]. Therefore, we test *“H1. Individuals with a stronger environmental identity experience higher levels of climate change worry*”.

Climate change literature testifies that, in moderation, worry contributes to adjusting and adapting to climate change [54,55]. López-García et al. [56] demonstrated that eco-worry mediated the relationship between willingness and climate agency with both sustainable consumption and active participation among 308 young adults. Behavioral theories such as the Protection Motivation Theory and the Health Belief Model suggest that worry or fear motivates adaptive behavior primarily when individuals also perceive that they are capable of taking effective action (i.e., when efficacy beliefs are high) [57–59]. However, the effects of worry are not linear. High levels of worry combined with low perceived efficacy or lack of clear behavioral guidance may lead to avoidance, disengagement, or reduced problem-solving capacity [60,61]. This helps explain why excessive worry can hinder adaptation efforts and lead to tension, distress, and decreased problem-solving abilities [62], and why empirical findings on eco-worry remain mixed. Thus, according to Bouman et al. [51], the existing evidence is insufficient to confirm whether worry can motivate people to take more specific actions to mitigate climate change. Similarly, Ágoston et al. [63] pointed out the diversity of findings about the consequences of eco-worry and anxiety (both stimulating and deterring action) and recommended further investigation. Recent research in environmental psychology similarly shows that climate concern is more likely to translate into pro-environmental action when accompanied by efficacy beliefs and actionable knowledge [64–66]. In response to this uncertainty, with the second hypothesis, we examined if “*H.2 Climate change worry positively predicts intention to act”.* Similarly, we expect that people who experience climate change worry are more likely to actively seek solutions, and therefore they are less bored with climate change. In this context, the following hypothesis stated that *“H3. The more people worry about climate change, the less likely they are to experience boredom with climate change”.* Boredom is a psychological state that implies the absence of meaningful tasks rather than the presence of stress [67], as happens in the case of climate change anxiety. Climate change boredom, similar to climate change skepticism, deserves attention since the individuals who experience it will not support environmentally friendly economic and political measures [68].

People with a solid environmental identity find climate change topics engaging and relevant [69]. Environmental identity reflects the degree to which environmental concerns are incorporated into one’s self-concept, influencing attention, motivation, and information processing. Individuals with a strong environmental identity are more likely to perceive climate change as personally meaningful, thereby sustaining cognitive engagement and reducing the likelihood of disengagement over time. Recent research shows that identity-congruent environmental information maintains attention and lowers psychological withdrawal, as it aligns with core values and reinforces self-consistency [70,71]. Because boredom emerges when an activity lacks meaning or fails to sustain attention, individuals who remain cognitively and emotionally engaged with climate change are less likely to experience boredom toward it. They may, therefore, perceive discussions about climate change as meaningful and important, which can lead to a lower likelihood of becoming bored with climate change. Consequently, we considered *“H4. Individuals with stronger environmental identity are less bored about climate change*”.

Environmental identity was often reported to be correlated with environmental factors, including climate change, values, behaviors, and actions [68,72]. Identity is becoming increasingly accepted as an essential driver of climate-related behavior [73]. From a theoretical perspective, identity-based motivation suggests that individuals are more likely to engage in behaviors that are congruent with their self-concept, as such actions reinforce a coherent sense of self. Empirical evidence indicates that environmental identity not only predicts pro-environmental intentions but also supports behavioral persistence over time, even in the absence of external incentives [e.g., [74,75]]. This implies that individuals who see themselves as environmentally responsible are more inclined to translate concern into concrete action, as acting against climate change becomes a self-expressive and norm-consistent behavior. Following this idea, we hypothesized that “*H5. Individuals with a stronger environmental identity are more prone to act against climate change*”.

Beliefs about timing, human-induced character, seriousness, and being a threat are defined by McCright et al. [76] as climate change beliefs. Individuals’ beliefs about climate change can impact their mitigation efforts [77] and their support of government actions [78]. In a meta-analysis of the determinants and outcomes of belief in climate change, Hornsey et al. [79] found that people who believed in climate change have stronger pro-environmental intentions. Romanian farmers who perceived a greater seriousness of climate change effects were more likely to adopt climate change adaptation measures [42]. An analysis by Berger and Wyss [80] of approximately 56,000 pollution-related decisions from over 2,200 participants across 30 countries revealed that belief in climate change strongly shaped decision-making. The authors showed that climate change skeptics tended to make self-serving choices, showing a tolerance for emissions even when the environmental impact was severe and personal gains were minimal. Despite the vast literature focused on beliefs related to climate change [81–83], considering that climate change beliefs differ between countries [84] and that there is still a research gap between understanding how climate change beliefs influence behavior and intention to act [85], we considered necessary to explore if Romanians’ beliefs in global climate change predict their intention to act. Hence, we examined whether *“H6. Belief in global climate change is positively associated with the intention to act”.* The following hypothesis – “*H7. Belief in global climate change is negatively associated with climate change boredom*” – is, to our knowledge, the first to investigate the potential negative correlation between climate change beliefs and climate change boredom. We assume that people who have strong beliefs about climate change are less likely to experience boredom. Beliefs in climate change could be associated with emotional responses such as concern, fear, or empathy for those affected by climate impacts [86,87]. These emotional connections can act as barriers to boredom because they provide personal and emotional reasons to remain engaged in climate change.

To explore the relationship between climate change goal commitment and boredom with climate change, we tested if “*H8. Climate change goal commitment is negatively associated with climate change boredom*”. Goal commitment can be defined as the cognitive and affective state characterized by the individual’s intention to strive to achieve a specific goal, the sustained pursuit of that goal over an extended period, and the resistance to reducing or abandoning the goal [88,89]. In the context of climate change, we understand climate change goal commitment as one’s dedication and willingness to act and make personal choices that help mitigate climate change and reduce one’s environmental impact. It involves a sense of responsibility and participation in addressing the challenges posed by climate change on an individual level. These commitments can manifest in various forms, such as consuming less, reducing energy consumption, recycling, or advocating for climate-friendly policies and behaviors [90–93].

It is important to recognize that the social context mediates emotional responses to climate change that are not based on personal experience [94]. Individuals tend to embrace beliefs, attitudes, and behaviors that align with the expectations of a particular social group [95]. Group membership allows individuals to have a sense of collective effectiveness, which refers to the belief that a group can achieve goals that exceed the capacities of its members [73,76]. When people say “We” instead of “I” to describe themselves, such as “members of an environmental action group”, or “members of a professional association,” they think of their actions as part of a more considerable group effort, which reduces the feeling of personal helplessness [96]. Forsyth et al. [97] reported greater willingness to protect water resources among those with a stronger sense of community, which aligns with other research in which feelings of belonging similarly influenced the desire to act [98,99]. Being part of a community that prioritizes environmental issues can give people emotional support, motivation, and a sense of power. Therefore, we explored “*H9. The feeling of belonging to a community that embraces climate change concerns is negatively associated with climate change boredom”.*

Self-efficacy of cooperation is another determinant for individuals and groups to engage in climate-friendly behaviors and actions [100]. Kerr [101] defines the “self-efficacy of cooperation” as the belief that an individual’s collaborative efforts play a significant role in the outcomes attained by a large group. Heath and Gifford [78] found that self-efficacy of cooperation was associated with the tendency to take more concrete steps against climate change. Similarly, the self-efficacy of cooperation was found to be, among others, an influential factor in the beliefs of Turkish undergraduate students about global climate change [102]. Yang et al. [103] found perceived self-efficacy to be a positive predictor of climate change adaptive action among 2,230 smallholder farmers and Sorour et al. [104] reported a significant positive correlation (r = 0.392) between environmental self-efficacy and pro-environmental behavior in 517 older adults. Engaging in actions that address climate change can give people a sense of efficacy and the belief that their efforts can make a difference. This belief in their efficacy can reinforce their intention to act. On the contrary, one’s trust in one’s ability to meaningfully contribute to group activities aimed at reducing climate change can be undermined by a lack of interest in climate change, motivation, or boredom. Hence, we tested: *“H10. The perceived self-efficacy of cooperation for climate change is negatively related to climate change boredom” and “H11. Self-efficacy of cooperation is positively related to the intention to act against climate change”.*

Climate change poses a multifaceted threat to public health, with rising temperatures, extreme weather events, and altered disease patterns contributing to increased health risks worldwide [105,106]. Vulnerable populations, including the elderly, children, and those with preexisting health conditions, are particularly susceptible to the adverse health effects of climate change, underscoring the urgent need for mitigation and adaptation strategies [45]. When people perceive climate change as a significant threat to their health, it tends to capture their attention and become a more salient issue in their lives. Halady and Rao [107] found that awareness of the health impact of climate change was the most significant factor that drove people to change their behavior and even advocate for environmental causes. Sun et al. [108] reported that a greater awareness of air pollution, influenced by an individual’s living conditions and health status, was strongly linked to heightened perceptions of climate change. Feeling that your health is at stake can provide a solid motivation to contribute to climate change mitigation efforts, reducing the likelihood of boredom. Considering the above, the next hypothesis reads as follows: “*H12. Higher perceived climate change influence on health leads to lower climate change boredom.*”

The role of demographics was found to be influential in many studies focused on climate change [109]. Poortinga et al. [84] showed that age influences climate change perceptions, with older people being more skeptical about the existence and anthropogenic source of climate change. Clayton et al. [4] examined demographic differences in emotional reactions to climate change. They found, for example, that gender matters, with women being more worried about climate change#39;s effects. Data analyses from data from 23 European countries revealed that climate change skepticism and concern were different between urban and rural areas, with higher climate change skepticism and lower concern for those living in a rural context [110]. The next hypothesis explores demographics: “*H13. Demographics have an impact on climate change boredom; H13a. The older people tend to be more bored about climate change; H13b. Men are more bored about climate change than women; H13c. People from urban areas are less bored about climate change.*”

Media, international organizations, and scientists occasionally worried that climate change boredom was in the background of inaction and failure to take meaningful actions against climate change [111]. Boredom also impairs the systematic use of learning strategies ([112], citing [113]), thus diminishing the efficiency of information-education programs. However, boredom did not receive much attention in the psychological literature until recently [18] and much less concerning climate change [111]. Possibly, the contrast between “the triviality of boredom and enormity of emergency and crisis” [111] moved the study focus away from climate change boredom. Geiger et al. [24] demonstrated that stronger boredom predicted decreased intention to engage in public-sphere climate action. We assumed that *“H14. Climate change boredom hinders the intention to act against climate change*”. Overall, the hypotheses are summarized in Table 1.

**Table 1:** Hypotheses of the study.

Number	Category and title
Antecedents and outcomes of environmental identity and climate change worry	
*H1*	*Individuals with a stronger environmental identity experience higher levels of climate change worry.*
*H2*	*Climate change worry positively predicts intention to act.*
*H3*	*The more people worry about climate change, the less likely they are to experience boredom with climate change.*
*H4*	*Individuals with stronger environmental identity are less bored about climate change.*
*H5*	*Individuals with a stronger environmental identity are more prone to act against climate change.*
Outcomes of beliefs: Beliefs about climate change as drivers of action and buffers against boredom	
*H6*	*Belief in global climate change is positively associated with the intention to act.*
*H7*	*Belief in global climate change is negatively associated with climate change boredom.*
Outcomes of efficacy and social factors	
*H8*	*Climate change goal commitment is negatively associated with climate change boredom.*
*H9*	*The feeling of belonging to a community that embraces climate change concerns is negatively associated with climate change boredom.*
*H10*	*The perceived self-efficacy of cooperation for climate change is negatively related to climate change boredom.*
*H11*	*Self-efficacy of cooperation is positively related to the intention to act against climate change.*
Contextual factors	
*H12*	*Higher perceived climate change influence on health leads to lower climate change boredom.*
*H13*	*Demographics have an impact on climate change boredom.* *H13a. The older people tend to be more bored about climate change; H13b. Men are more bored about climate change than women; H13c. People from urban areas are less bored about climate change.*
Climate change boredom as an inhibitor of action	
*H14*	*Boredom is negatively related to the intention to act against climate change.*

This set of hypotheses reflects a theoretically informed model in which climate change boredom is positioned as a central outcome of multiple psychological and social antecedents and as a key inhibitor of behavioral intentions. By explicitly integrating emotional, cognitive, and social dimensions, the proposed framework extends existing approaches and provides a more comprehensive understanding of the mechanisms underlying climate change action.

## 3. Methodology

### 3.1. Study area, target population, and data collection

The study was conducted in Romania, targeting the adult population aged 18 and over. Data were collected through a professional online market research company operating a large-scale consumer panel, which ensured access to a diverse pool of respondents and facilitated demographic representativity. Participants were recruited from an existing opt-in panel of individuals who had previously agreed to participate in survey-based research and were invited via email to take part in the study. In exchange for their response, participants received reward points within the panel system, which could be redeemed for goods, vouchers, or discounts from affiliated partners. The questionnaire was administered online by the specialized company to 813 Romanian respondents (01.11.2023–10.11.2023). The sample is representative at the country level for age, gender, and region. Respondents’ profile is included in Table 1A, Appendix. Participation was voluntary, and informed consent was obtained from all participants before starting the survey, describing the purpose of the research, the expected duration, and the voluntary nature of their participation. Anonymity was ensured as the respondents’ identities were not linked to their responses. The research was conducted with the utmost respect for the ethical principles of research integrity (Ethical approval certification no. 8819/ 30.06.2023, issued by Babes-Bolyai University, Romania).

### 3.2. Research instrument development

The research instrument was developed by integrating multiple validated scales from prior literature into a single structured questionnaire. Each construct included in the conceptual model was operationalized using previously established measures, which were adapted where necessary to fit the context of climate change. All items were harmonized in terms of wording and response format to ensure consistency and comparability across constructs.

The revised scale of the environmental identity of Clayton et al. [46] was used. We implemented the 10-item climate change worry scale developed by Stewart et al. [114] to investigate climate change worry. Beliefs about climate change were investigated across three dimensions: the existence of climate change, its causes, and its gravity. For climate change goal commitment, we used the nine-item commitment scale of Hollenbeck et al. [115], which was adapted to climate change. To measure boredom, nine items from Thompson and Barton’s [116] study, in which environmental apathy was investigated, were adapted to our focus on climate change. Apathy and boredom are distinct constructs, but both involve disengagement and lack of interest. Apathy reflects a more generalized indifference, while “feeling unchallenged” is central to boredom [117], which is also a temporary state of dissatisfaction arising from a lack of stimulation or meaningful engagement [118]. These overlapping characteristics suggest that the items may capture certain aspects of disengagement relevant to climate change boredom, even though they were not explicitly developed for this construct.

Four statements from Heath and Gifford [78] explored the self-efficacy of cooperation. The intention to act against climate change can encompass a variety of actions, such as recycling, advocating for environmental policies, or participating in environmental activism [119,120]. This diversity led us to include two categories of items to measure the intention to act. One focused on general behavior (for example, the intention to take specific measures to fight climate change) and contained four statements from Heath and Gifford [78]. The other asked about six particular behaviors, such as the intention to reuse and recycle more. They were selected based on a pre-test that asked 149 people to indicate from a pre-defined list what behaviors could be the most effective in reducing climate change. Two questions examined the perceived influence of climate change on health. They provide information on how respondents perceive the effect of climate change on their current health condition and how they believe climate change will influence their health in the future. Demographics analyzed here included age, gender, and living environment. All variables, their items, and answer options in the questionnaire are presented in Table 1A, Appendix.

### 3.3. Data analysis

Given that all variables were latent variables, the structural equation modeling (SEM) technique was used to test the proposed hypotheses and their causal relationships. There are two approaches to examining structural equation models: the covariance-based approach (CB-SEM) and the variance-based partial least squares approach (PLS-SEM) [121]. This study used PLS-SEM to test the proposed hypotheses and their causalities. This choice can be justified for several reasons. First, compared to CB-SEM, PLS-SEM does not require the removal of many indicators to achieve an acceptable model fit [122]. Second, PLS-SEM is not constrained by sample size restrictions or assumptions of normal data distribution, which is rarely met in social research. Lastly, CB-SEM is typically used for confirmatory research and confirming established theories, while PLS-SEM is more suitable for exploratory research. Therefore, the use of PLS-SEM is reasonable and appropriate [121].

**Fig 2:**
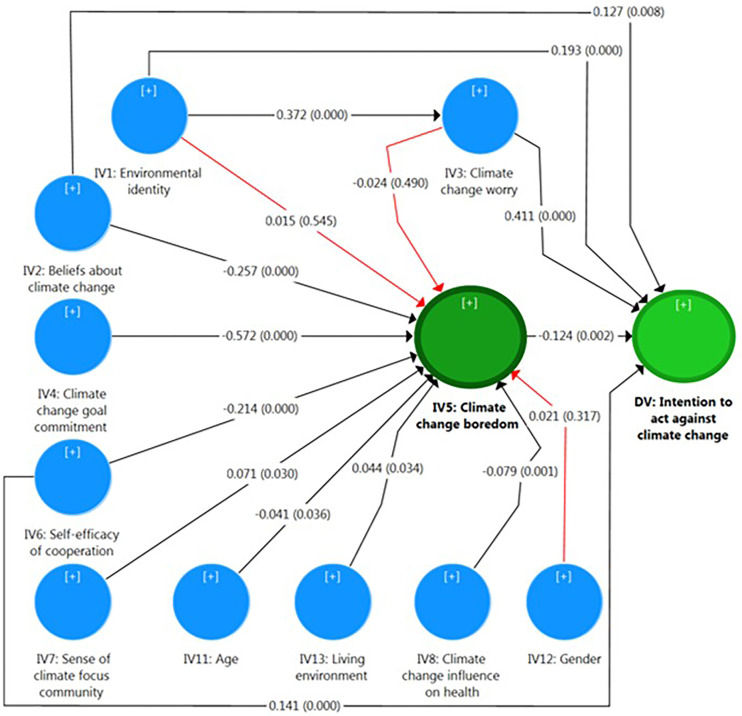
Direct effects (on arrows) and significant level (in parentheses) of variables.

The analysis was conducted in a stepwise manner. First, the measurement model was assessed through confirmatory factor analysis, including the evaluation of indicator reliability (factor loadings), internal consistency reliability (Cronbach’s alpha and composite reliability), and convergent validity (average variance extracted) (as reported in the Results section, subsection 4.1, Table 2). Discriminant validity was subsequently examined using the heterotrait–monotrait ratio (HTMT), that is, the ratio of the between-trait correlations to the within-trait correlations (subsection 4.1., Table 3). Second, after establishing the adequacy of the measurement model, the structural model was evaluated by estimating path coefficients and their significance levels (sub-section 4.2., Fig 2), along with the calculation of total effects (sub-section 4.2., Table 4) to capture both direct and indirect relationships. Finally, the predictive power and model quality were assessed using coefficients of determination (R^2^ and adjusted R^2^) and predictive relevance (Q^2^ based on SSO and SSE) (sub-section 4.2., Table 5).

**Table 2:** Factor loadings, AVE, CR, Cronbach’s alpha of the constructs.

Construct Item	Factor loading	Cronbach alpha ofconstruct	CR	AVE
DV: Intention to act against climate change		0.888	0.913	0576
Q6_11_3	0.758			
Q6_12_3	0.660			
Q6_13_3	0.772			
Q6_14_3	0.803			
Q6_5_2	0.843			
Q6_6_2	0.440			
Q6_7_2	0.853			
Q6_8_2	0.853			
IV1: Environmental identity		0.958	0.962	0.662
Q1_10	0.826			
Q1_11	0.868			
Q1_12	0.780			
Q1_13	0.864			
Q1_14	0.815			
Q1_2	0.786			
Q1_3	0.787			
Q1_4	0.833			
Q1_6	0.807			
Q1_7	0.795			
Q1_8	0.822			
Q1_9	0.845			
Q1_1	0.735			
IV2: Beliefs about climate change		0.917	0.932	0.604
Q2_11_3	0.785			
Q2_12_3	0.782			
Q2_1_1	0.810			
Q2_2_1	0.852			
Q2_3_1	0.740			
Q2_4_1	0.817			
Q2_5_1	0.827			
Q2_7_2	0.676			
Q2_9_2	0.690			
IV3: Climate change worry		0.961	0.966	0.738
Q3_1	0.841			
Q3_10	0.875			
Q3_2	0.892			
Q3_3	0.800			
Q3_4	0.902			
Q3_5	0.830			
Q3_6	0.844			
Q3_7	0.863			
Q3_8	0.915			
Q3_9	0.823			
IV4: Climate change goal commitment		0.829	0.876	0.588
Q4_1	0.691			
Q4_2	0.715			
Q4_4	0.835			
Q4_6	0.743			
Q4_9	0.836			
IV5: Climate change boredom		0.935	0.946	0.689
Q5_1	0.832			
Q5_2	0.834			
Q5_3	0.823			
Q5_4	0.814			
Q5_6	0.828			
Q5_7	0.819			
Q5_8	0.833			
Q5_9	0.855			
IV6: Self-efficacy of cooperation		0.807	0.911	0.837
Q6_3_1	0.931			
Q6_4_1	0.898			
IV7: Sense of climate-focused community		0.946	0.951	0.682
Q7_1	0.676			
Q7_2	0.864			
Q7_3	0.852			
Q7_4	0.874			
Q7_5	0.852			
Q7_6	0.833			
Q7_7	0.775			
Q7_8	0.843			
Q7_9	0.847			
IV8: Climate change influence on health		0.647	0.843	0.731
Q8_1	0.920			
Q8_2	0.784			
IV11: Age	1	1	1	1
IV12: Gender	1	1	1	1
IV13: Living environment	1	1	1	1

**Table 3:** Discriminant validity.

	1	2	3	4	5	6	7	8	9	10	11
1. DV: Intention to act against climate change											
2. IV11: Age	0.064										
3. IV12: Gender	0.084	0.016									
4. IV13: Living Environment	0.036	0.052	0.013								
5. IV1: Environmental identity	0.467	0.135	0.088	0.024							
6. IV2: Beliefs about climate change	0.592	0.062	0.068	0.033	0.449						
7. IV3: Climate change worry	0.623	0.034	0.098	0.024	0.362	0.697					
8. IV4: Climate change goal commitment	0.248	0.133	0.064	0.026	0.149	0.159	0.191				
9. IV5: Climate change boredom	0.396	0.163	0.094	0.051	0.234	0.373	0.164	0.789			
10. IV6: Self-efficacy of cooperation	0.295	0.088	0.027	0.021	0.145	0.093	0.055	0.698	0.663		
11. IV7: Sense of climate-focused community	0.387	0.103	0.129	0.032	0.19	0.146	0.25	0.119	0.087	0.039	
12. IV8: Climate change influence on health	0.457	0.12	0.109	0.059	0.184	0.554	0.607	0.112	0.308	0.143	0.129

**Table 4:** Total effects of the independent variables.

Path (from → to)	Total effects	St. Dev.	P Values
IV1: Environmental identity → DV: Intention to act against climate change	0.345	0.037	0.001
IV1: Environmental identity → IV3: Climate change worry	0.372	0.034	0.001
IV1: Environmental identity → IV5: Climate change boredom	0.007	0.027	0.810
IV2: Beliefs about climate change → DV: Intention to act against climate change	0.159	0.046	0.001
IV2: Beliefs about climate change → IV5: Climate change boredom	−0.257	0.031	0.001
IV3: Climate change worry → DV: Intention to act against climate change	0.414	0.039	0.001
IV3: Climate change worry → IV5: Climate change boredom	−0.024	0.034	0.489
IV4: Climate change goal commitment → DV: Intention to act against climate change	0.071	0.023	0.002
IV4: Climate change goal commitment → IV5: Climate change boredom	−0.572	0.030	0.001
IV5: Climate change boredom → DV: Intention to act against climate change	−0.124	0.039	0.002
IV6: Self-efficacy of cooperation → DV: Intention to act against climate change	0.168	0.035	0.001
IV6: Self-efficacy of cooperation → IV5: Climate change boredom	−0.214	0.030	0.001
IV7: Sense of climate-focused community → DV: Intention to act against climate change	−0.009	0.005	0.091
IV7: Sense of climate-focused community → IV5: Climate change boredom	0.071	0.032	0.026
IV8: Climate change influence on health → DV: Intention to act against climate change	0.01	0.005	0.038
IV8: Climate change influence on health → IV5: Climate change boredom	−0.079	0.025	0.001
IV11: Age → DV: Intention to act against climate change	0.005	0.003	0.079
IV11: Age → IV5: Climate change boredom	−0.041	0.020	0.035
IV12: Gender → DV: Intention to act against climate change	−0.003	0.003	0.357
IV12: Gender → IV5: Climate change boredom	0.021	0.021	0.324
IV13: Living Environment → DV: Intention to act against climate change	−0.005	0.003	0.080
IV13: Living Environment → IV5: Climate change boredom	0.044	0.020	0.031

**Table 5:** Values for identifying dependent constructs.

Construct	SSO	SSE	Q² (=1-SSE/SSO)	R^2^	R^2^_adj._
DV: Intention to act against climate change	6,504.00	4,882.68	0.249	0.475	0.471
IV3: Climate change worry	8,130.00	7,388.49	0.091	0.138	0.137
IV5: Climate change boredom	6,504.00	3,723.49	0.428	0.668	0.664

## 4. Results

### 4.1. Sample characteristics and descriptive statistics

The final sample consisted of 813 respondents, of which 51.8% were female and 48.2% male. The mean age of participants was 45.6 years (SD = 13.3). Regarding living environment, 88.7% resided in urban areas and 11.3% in rural areas. The sample is representative of the Romanian adult population in terms of age, gender, and region, based on quotas applied during data collection. The response rate was 35% (calculated based on the number of completed questionnaires relative to the number of panel invitations sent).

### 4.2. Confirmatory factor analysis (CFA), construct reliability and validity, and the fit model

The standardized root mean square residual (SRMR) and Normed Fit (NFI) measures were used to assess the fit between the model and the data. Model fit was assessed using SRMR and NFI, following established thresholds reported in the methodology section. Specifically, SRMR must be equal or lower than 0.08, and NFI must be equal or higher than 0.9. In our study SRMR = 0.05 and NFI = 0.90. These values indicate a good model fit. We used confirmatory factor analysis (CFA) to assess the reliability and validity of the research model. Table 2 presents factor loadings, average variance extracted (AVE), combined reliability (CR), and Cronbach’s alpha of the constructs. All of these indicators met the necessary criteria for further analysis to proceed [121] (Table 2). Items with factor loadings less than 0.4 were removed [123] (they are marked with “#” in S1 Table, Supplementary material).

Table 3 shows heterotrait-monotrait ratio (HTMT) of the correlations. An HTMT value above 0.90 suggests a lack of discriminant validity. All HTMT values in this study are below the recommended threshold of 0.90, indicating that the constructs are empirically distinct. When the constructs in the path model are conceptually more distinct, a lower and thus more conservative threshold value of 0.85 seems warranted [121]. Table 3 shows that the coefficients of each variable do not interact with each other. These results confirm that discriminant validity is satisfactorily established for all variables included in the model.

### 4.3. Evaluation of research hypotheses and total effects

The PLS-SEM approach was used to evaluate research hypotheses. Fig 2 shows the path coefficients and their significance levels, while Table 4 reports the total effects of the research model. As shown in Fig 2, 13 out of the 16 hypothesized direct relationships are statistically significant (black arrows), whereas three paths are not significant (red arrows).

#### 4.3.1. Antecedents and outcomes of environmental identity and climate change worry (H1-H5).

According to the results, environmental identity positively and significantly affects climate change worry (β = 0.372, sig. = 0.001; H1). Climate change worry (β = 0.411, sig. = 0.000; H2) and environmental identity (β = 0.193, sig. = 0.001; H5) have a positive and significant influence on the intention to act against climate change. Climate change worry (H3) and environmental identity (H4) do not significantly affect climate change boredom.

#### 4.3.2. Outcomes of beliefs: Beliefs about climate change as drivers of action and buffers against boredom (H6, H7).

Beliefs about climate change (β = 0.127, sig. = 0.009; H6) have a positive and significant influence on the intention to act against climate change. Beliefs about climate change (β = −0.257, sig. = 0.001; H7) have a negative and significant effect on climate change boredom.

#### 4.3.3. Outcomes of efficacy and social factors (H8-H11).

Climate change goal commitment (β = −0.572, sig. = 0.001; H8) and self-efficacy of cooperation (β = −0.214, sig. = 0.001; H10) have a negative and significant effect on climate change boredom. The sense of climate-focused community (β = 0.071, sig. = 0.030; H9) positively and significantly affects climate change boredom. Self-efficacy of cooperation (β = 0.141, sig. = 0.001; H11) has a positive and significant influence on the intention to act against climate change.

#### 4.3.4. Contextual factors (H12, H13).

Climate change effect on health (β = −0.079, sig. = 0.002; H12), and age (β = −0.041, sig. = 0.036; H13a) have a negative and significant effect on climate change boredom. The living environment (β = 0.044, sig. = 0.032; H13c) has a positive and significant effect on climate change boredom. Gender (H13b) does not significantly affect climate change boredom.

#### 4.3.5. Climate change boredom as an inhibitor of action (H14).

**Fig 3:**
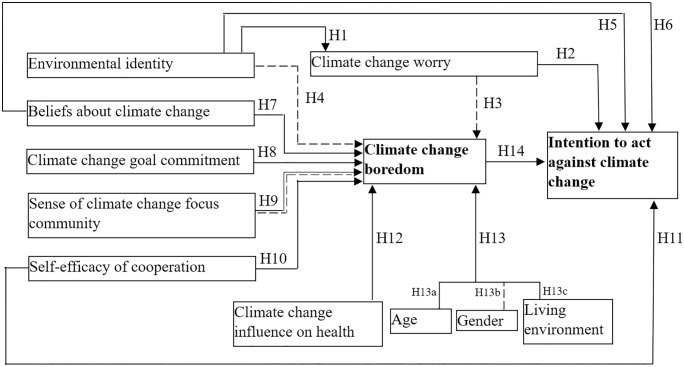
Results regarding the validation of research hypotheses [guide for hypothesis type: continuous line = confirmed; dashed line = not confirmed; continuous and dashed line = partially confirmed (H9; the relationship exists, but the direction is the opposite to the hypothesized one)].

Climate change boredom (β = − 0.124, sig. = 0.002) negatively influences the intention to act against climate change (H14). Fig 3 presents an overview of the confirmation of the research hypothesis.

Regarding the structural model, the PLS-based SEM procedure was utilized to analyze Stone-Geisser#39;s Q^2^, R^2^, and path coefficients. This analysis evaluated the predictive accuracy and strength of the relationship between variables in the defined paths. As presented in Table 5, the Q^2^ values, representing cross-validated redundancy for the endogenous variables of the model, were all positive, ranging from 0.091 to 0.428. This suggests that the model exhibits reliable predictive accuracy. Furthermore, the total adjusted R^2^ was 0.475, indicating that the model possessed a suitable moderate predictive power. Overall, the model shows that climate change boredom, environmental identity, beliefs about climate change, climate change worry, and self-efficacy of cooperation predict 47.5% of the intention to act against climate change (Table 5). Also, beliefs about climate change, climate change goal commitment, self-efficacy of cooperation, sense of climate-focused community, age, living environment, and climate change influence on health predict 66.8% of climate change boredom (Table 5).

## 5. Discussion

This study advances the emerging literature on climate emotions by positioning climate change boredom as a central psychological barrier within a broader cognitive–affective–social framework. Grounded in behavior change theory, particularly the Theory of Planned Behavior, the findings suggest that behavioral intention is shaped not only by cognitive beliefs and perceived control, but also by affective and identity-based processes that influence engagement. While prior research has largely focused on anxiety, fear, or hope, our findings confirm that boredom is not merely a passive state but a functionally significant predictor of disengagement, directly reducing the intention to act. This aligns with recent evidence showing that boredom signals insufficient meaningful engagement and redirects attention away from goal-relevant behavior [18,24]. Moreover, consistent with Protection Motivation Theory, the results indicate that emotional responses alone are insufficient to motivate action unless accompanied by efficacy-related and goal-oriented factors. By explaining 47.5% of the variance in intention and 66.8% in boredom, the model demonstrates substantial explanatory power and highlights boredom as a critical, but underexplored, mechanism in climate inaction.

### 5.1. Antecedents and outcomes of environmental identity and climate change worry (H1-H5)

The results indicate that *“H1. Individuals with a stronger environmental identity experience higher levels of climate change worry*”, *“H2. Climate change worry predicts the intention to act*” and “*H5. Individuals with stronger environmental identity are more prone to act against climate change*” are confirmed. This means that environmental identity has both a direct and indirect (through climate change worry) effect on the intention to act, suggesting the possibility of using both paths to strengthen the intention to act. This pattern is theoretically important because it indicates that environmental identity is not only a value orientation, but also a motivational resource that activates emotional responsiveness and action readiness. H1 shows that people who strongly identify with environmental values and consider them an integral part of their identity are more likely to worry about climate change and be more attuned to its consequences, and, according to H2, this leads to the intention to act. In other words, when climate change is perceived as a threat to something central to the self, emotional engagement becomes more likely, and this engagement can be translated into behavioral intention. H2 highlights the motivational aspect of worry in the context of climate change. When people are worried about climate change, they are more likely to feel a moral obligation to act. This aligns with previous research indicating that emotional responses like worry and fear can predict climate change action [124,125]. Bouman et al. [51] and Szabó and Lovibond [126] demonstrated that those who worry about a real or imagined threat are more likely to take action to deal with the threat. Our findings, therefore, support the argument that worry should not be interpreted exclusively as a maladaptive response. Under the right psychological conditions, it can function as an activating emotion that keeps climate change cognitively salient and behaviorally relevant, which is consistent with evidence showing that climate risk perceptions are shaped not only by cognition but also by affective processes that support engagement [66] and with research showing that some climate-related emotions can motivate action when they are linked to a sense of response relevance and agency.

Such an outcome underscores the potential for emotional appeals in climate change communication and activism. However, this potential should be used within proper limits because studies showed that worry determines adaptive behavior only when the situation is seen as controllable [52,127]. Worry can cause stress and low well-being in uncontrollable cases and cannot lead to constructive efforts. Thus, the present results suggest that climate communication should not aim simply to intensify worry, but to pair worry with feasible pathways for action, efficacy cues, and concrete opportunities for engagement.

The confirmation of H5 underscores the opportunity to leverage environmental identity to mobilize collective action against climate change. Previous research demonstrated the link between identity and behavior [75,128,129]. Thus, encouraging individuals to cultivate and strengthen their environmental identities can be an effective strategy for building a more sustainable society. Importantly, the direct effect of environmental identity on intention to act suggests that identification with environmental values may guide action even beyond specific emotional states. This reinforces the idea that identity-based interventions may be particularly powerful because they can influence both how people interpret climate threats and how they define appropriate responses. This aligns with the broader imperative of environmental security, which emphasizes the need to maintain ecological stability to prevent conflicts and disruptions caused by environmental degradation [77].

Contrary to our expectations, “*H3. The more people worry about climate change, the less likely they are to experience climate change boredom*” and “*H4. Individuals with stronger environmental identity are less bored with climate change*” were not confirmed by the PLS-SEM analyses It is worth noting that although worry and environmental identity do not lead to boredom (H3 and H4 not confirmed), they increase the intention to act (H2 and H5 confirmed). This is one of the most theoretically interesting findings of the study because it suggests that boredom is not simply the opposite of concern. People may care about climate change and still become bored by how the issue is encountered, framed, or repeated in everyday life, which is in line with the Meaning and Attentional Components model of boredom, according to which boredom emerges when an activity fails to sustain attention or lacks perceived meaning [18].

For H3, an explanation could stand in the fact that worry and boredom may not share a common psychological mechanism and may not influence each other in a consistent way [130]. Climate change worry and boredom are not static or fixed responses but dynamic and context-dependent ones. Possibly, climate change worry and climate change boredom are not directly related, but could be rather mediated by other factors, such as perceived efficacy. For example, some people may cope with their climate change worry by engaging in positive actions, such as recycling, joining environmental movements, or educating others about the issue. These actions may increase their sense of agency and hope [24] and reduce their boredom. On the other hand, some people may cope with their worry by avoiding or denying the problem, which may lead to feelings of helplessness and increase their boredom. Therefore, the relationship between worry and boredom may depend on how people manage their emotions and on their beliefs about climate change. A plausible interpretation is that worry reflects emotional arousal, whereas boredom reflects insufficient meaning, insufficient challenge, or repetitive exposure. As a result, a person may be emotionally concerned but not cognitively engaged. This interpretation is also consistent with findings showing that, when people think about climate action, boredom predicts lower action intentions even when other negative emotions are present, indicating that boredom is a distinct disengagement pathway rather than merely a weaker form of concern [24].

One explanation why environmental identity does not predict climate change boredom (i.e., H4 not confirmed) could be that environmental identity is a multifaceted construct that may not capture the specific motivations and emotions relevant to climate change. Similar results report limitations of environmental identity as a predictor, for example, of climate change attitudes and behaviors. Fielding et al. [131] found that environmental identity was positively related to pro-environmental behavior, but only when it was aligned with other social identities, such as political orientation or group membership. They argued that environmental identity is not a static or consistent concept but rather a dynamic and context-dependent one that can fluctuate based on the situation and the prominence of other identities. Our findings are consistent with this interpretation and suggest that environmental identity may be sufficient to stimulate concern and action intention, but insufficient on its own to protect individuals from disengagement. Preventing boredom may require not only identification with environmental values, but also novelty, efficacy, and personally meaningful forms of participation.

### 5.2. Outcomes of beliefs: Beliefs about climate change as drivers of action and buffers against boredom (H6, H7)

Both hypotheses related to beliefs about climate change were confirmed: *“H6. Belief in global climate change is positively associated with intention to act*” and *“H7. Belief in global climate change is negatively associated with climate change boredom*”. These findings reveal the importance of public belief in climate change as a driver of action. Beliefs in climate change serve as a foundational step in the behavioral change process. People who acknowledge the reality of climate change (its existence, causes, and gravity) are more likely to feel a sense of responsibility and urgency to act. For example, in the context of reducing meat consumption as a climate change mitigation strategy, the belief in the severity of climate change was a strong predictor of mitigation intentions [132]. Based on survey data, Brügger et al. [133] found that people in the UK and Switzerland who thought climate change was real and regarded it as a risk were ready to do something about it. The present study extends this line of argument by showing that climate change beliefs are not only linked to action-oriented intentions but also to lower boredom. This indicates that belief strength may help maintain the salience and relevance of the issue over time.

Similarly, H7 showed that climate change boredom feelings were less prevalent among interviewed people who strongly believed in climate change. This is a promising finding, as it implies that fostering beliefs about climate change can prevent people from being bored. Lack of boredom can reduce the risk of disconnecting from this critical issue, as it was observed that boredom may signal disengagement [19]. From a practical perspective, this means that communication efforts aimed at strengthening belief in reality, seriousness, and human causes of climate change may have a double benefit: they may increase willingness to act and simultaneously reduce motivational withdrawal from the issue.

### 5.3. Outcomes of efficacy and social factors (H8-H11)

The negative association between climate change goal commitment and climate change boredom (hypothesized in “*H.8 Climate change goal commitment is negatively associated with climate change boredom*”) underscores the importance of individual motivation in addressing climate change. Zhang and Huang [134] consider that one’s commitment to reach a goal depends on the degree to which one person believes the goal is attainable. This finding highlights the need for interventions and strategies to revitalize motivation and commitment among people experiencing boredom with climate change and to foster trust in people’s capacity to contribute to attaining this goal. Conceptually, this result suggests that boredom is reduced when climate change is framed not only as a threat, but also as a goal-directed domain in which personal effort has direction and purpose. When individuals remain committed to reducing climate change, the issue is less likely to be perceived as futile, repetitive, or psychologically empty.

The positive association between the sense of a climate change-focused community that embraces climate change concerns and climate change boredom is interesting. Thus, “*H9. The sense of climate-focused community that embraces climate change concerns is negatively associated with climate change boredom*” was partially confirmed: the relation exists, but its direction differs from the hypothesized one (positive instead of negative). In the present case, the more connected people feel to a climate-focused community, the more bored with climate change they are. This counterintuitive result deserves careful attention because it suggests that social embeddedness does not automatically produce motivational benefits. In some cases, repeated exposure to similar discussions, messages, or concerns within one’s community may generate message fatigue rather than sustained engagement.

Also, the community’s low concern for climate change may detach people from this problem and lead to boredom. Respondents rated average scores on the items related to climate change concerns within the scale for a sense of climate-focused community (items 2, 5, and 6, Table 1A, Appendix). Another possible explanation is that belonging to a climate-focused community may increase awareness of the problem without necessarily increasing perceived progress or efficacy. Under such conditions, belonging may heighten exposure but not empowerment, potentially leading to disengagement rather than activation. Future research should therefore investigate not only whether climate-focused communities exist, but also how they communicate, how often climate issues are discussed, whether discussions are solution-oriented, and whether participants perceive these communities as energizing or exhausting.

“*H10. Perceived self-efficacy of cooperation for climate change is negatively related to climate change boredom*” and *“H11. Self-efficacy of cooperation is positively related to the intention to act against climate change*” were confirmed by statistical analyses. The negative relationship between the perceived self-efficacy of cooperation for climate change and climate change boredom (H10) indicates that those who doubt their ability to contribute effectively to climate action through collaboration experience boredom. This is congruent with other results reported in the literature, demonstrating that self-efficacy is a dispositional control appraisal that may reduce boredom [135]. The perceived lack of self-efficacy of cooperation means it is impossible or difficult to achieve a helpful outcome through joint efforts, which makes cooperation pointless in people’s eyes. This interpretation is particularly important because it indicates that boredom may emerge when people perceive a disconnect between effort and outcome. If individuals believe that cooperative action will not matter, disengagement becomes a psychologically understandable response.

The positive correlation between self-efficacy of cooperation and the intention to act against climate change (H11) reaffirms the importance of self-belief in fostering pro-environmental behaviors. Individuals who feel capable of cooperating with others to address climate change are more likely to express a genuine intention to act. Similarly, O’Connor and Keil [136] confirmed that individuals were more inclined to engage in environmental initiatives when they perceived a higher level of organizational efficacy, which could be achieved through cooperation. Together, H10 and H11 suggest that efficacy beliefs do more than stimulate action; they also help protect against disengagement. This makes self-efficacy of cooperation a particularly valuable target for intervention through climate communication, education, and community mobilization.

### 5.4. Contextual factors (H12, H13)

The confirmation of “*H12. Higher climate change influence on health leads to lower climate change boredom*” reflects that individuals who perceive climate change as a tangible threat to their health are more likely to remain engaged and active in addressing this global challenge. The connection between health and engagement with climate change indicates that public health campaigns and educational initiatives play a significant role in maintaining people#39;s interest and motivation to act against climate change. According to Meyer et al. [62], framing climate change as a potential public health threat generates emotional reactions that can support endeavors aimed at reducing and adapting to the impacts of climate change.

This finding suggests that proximity matters. When climate change is perceived through concrete personal consequences, especially health-related ones, the issue becomes harder to dismiss, ignore, or experience as abstract and repetitive. In this sense, health framing may be one of the most promising communication strategies for counteracting boredom and strengthening engagement.

In this study, stronger climate change boredom was observed among older people and those from rural areas, but it was not influenced by gender. Older people and rural ones are important demographic groups because the population is aging [137], and almost half of the country’s population lives in rural areas [47.7% rural population, in 2022, according to [137]]. It is possible that, as one gets older, the meaning of the future changes becoming less important [138], citing [139,140] and, as climate change is a problem with implication for the future, may be less important for them and become boring. A change in people’s norms regarding environmental concern and growing environmental education in the curriculum were mentioned to be reasons for higher environmental concern among younger people (lower for older) [141]. This could also be the case for climate change boredom. These findings suggest that boredom is also socially and contextually patterned, individually experienced. Climate change may be perceived differently depending on life stage, lived experience, and the perceived relevance of the issue to everyday life.

Boredom may be more present in rural areas because here, people have less opportunity to take efficient measures to prevent it and to respond to its effects [141], and the lack of successful outcomes may drive people to disconnect from this issue a coping response to this unpleasant feeling and generate boredom. Whitmarsh [141] citing [142] mentioned that lower environmental concern among rural people may appear because nature is more likely to carry an instrumental than symbolic meaning for rural communities. Messages about climate change should better consider the characteristics of older people and rural inhabitants. To decrease boredom, messages should focus on aspects that have the capacity to capture their attention and are meaningful to them, because it was demonstrated that boredom has two components: an attentional (i.e., able to) one and a meaning (i.e., want to) one [18]. Hornsey and Lewandowsky [143] found that climate change skeptics are more influenced by messengers with similar identities, which may imply that rural people will perceive a message from rural people as more attractive (less boring), and older people will react better to older peers. Accordingly, climate communication should move beyond one-size-fits-all approaches and use audience-sensitive framing, trusted messengers, and locally meaningful examples to reduce boredom and strengthen relevance.

### 5.5. Climate change boredom as an inhibitor of action (H14)

The study results confirmed that boredom prevents people from taking action on climate change (*H14*). Naturally, people will seek a way to reduce boredom, and there are four primary routes for this – switching activities, regulating goal value, regulating cognitive demand, and regulating mental resources – with the first seeming to be the most effective [18]. Danckert and Eastwood [144] highlight that boredom is “a call to action, a signal to become more engaged” and redirects people’s efforts to other more meaningful and rewarding activities. Furthermore, Westgate and Wilson [18] named boredom “an immensely powerful motivator of people’s actions, for both better and worse” [from bursts of creativity to self-destructive drug use) because it encourages people to take action toward regaining successful engagement in a meaningful activity [18]]. In the context of climate change, this means boredom does not imply emotional neutrality. Rather, it signals that attention and motivation are likely to be redirected elsewhere, a pattern that accords with both the functional account of boredom advanced by Westgate and Wilson [18] and with climate-specific evidence showing that boredom is associated with weaker intentions to take climate action [24].

When people fail to alleviate boredom on their own, external factors can help. To efficiently prevent people from becoming bored about climate change or reduce their boredom to strengthen their intention to act against climate change, it is essential to find out what leads to boredom. This study showed that people with increased environmental identity, who believe in climate change, are committed to the goal of reducing it, believe in the self-efficacy of cooperation, perceive a higher impact of climate change on health, and are less bored about climate change. The practical implication is that anti-boredom strategies should not focus only on attention-grabbing messages, but also on restoring meaning, efficacy, and perceived attainability. Climate communication is likely to be more effective when it combines credible risk information with clear solutions, collective efficacy, and personally relevant reasons to stay engaged. Addressing boredom is crucial to maintaining long-term engagement and preventing people from burning out. In other research fields, boredom and burnout were shown to be positively and reciprocally related [145].

Building on these empirical findings, the results also generate important theoretical and policy implications that further clarify the role of climate change boredom within the broader framework of climate-related behavior

### 5.6. Theoretical and policy implications

The present study offers theoretical contributions by advancing current understanding of the psychological mechanisms underlying climate action. Anchored in behavior change theory, particularly the Theory of Planned Behavior, the findings extend existing models by demonstrating that behavioral intention is influenced not only by cognitive beliefs and perceived control, but also by affective and identity-based processes that shape engagement. The findings position climate change boredom as a central construct within cognitive-affective models, demonstrating that it is not merely the absence of concern but a distinct disengagement pathway that significantly reduces intention to act (β = −0.124, *p* = 0.002). At the same time, worrying about climate change positively predicts intention (β = 0.411, *p* < 0.001) but does not reduce boredom, indicating that engagement and disengagement are driven by different psychological processes rather than representing opposite ends of a single continuum. This supports a more nuanced framework in which individuals may simultaneously experience concern and disengagement, with different emotional mechanisms shaping behavioral outcomes.

Furthermore, the results extend motivational and attentional theories by showing that boredom is strongly influenced by goal-related and efficacy-related factors. Consistent with Protection Motivation Theory, these findings highlight that emotional arousal alone is insufficient to sustain engagement unless individuals perceive their actions as meaningful and effective. Climate change goal commitment (β = −0.572) and self-efficacy of cooperation (β = −0.214) significantly reduce boredom, suggesting that disengagement emerges when climate action lacks perceived meaning, direction, or attainability. In addition, the unexpected positive association between a sense of climate-focused community and boredom (β = 0.071) challenges assumptions about the uniformly beneficial role of social context, suggesting that repeated exposure or message homogeneity may lead to disengagement. Overall, the findings contribute to theory by integrating emotional, cognitive, and social dimensions into a dual-path framework of engagement (driven by identity, worry, and efficacy) and disengagement (driven by boredom and reduced perceived meaning).

In addition to these theoretical contributions, the findings provide several policy implications for improving the effectiveness of climate strategies and communication:

i) Climate policies should move beyond awareness-raising approaches and focus on sustaining engagement over time, as individuals may remain informed yet disengaged due to boredom.
ii) Interventions should strengthen climate change goal commitment by translating abstract objectives into concrete, achievable actions and by providing visible indicators of progress.
iii) Communication strategies should enhance perceived self-efficacy of cooperation by emphasizing the impact of collective action and demonstrating how individual contributions lead to meaningful outcomes.
iv) Framing climate change in terms of immediate and personally relevant consequences, particularly health-related impacts, can help reduce boredom and maintain attention.
v) Community-based approaches should avoid repetitive or homogeneous messaging and instead incorporate diverse, solution-oriented, and participatory formats to prevent message fatigue.
vi) Last but not least, targeted strategies are needed for groups more prone to boredom, such as older individuals and rural populations, using locally relevant examples and trusted messengers.

Overall, the results suggest that effective climate policies should not only aim to increase concern but also explicitly address the psychological drivers of disengagement to ensure sustained motivation and participation in climate action.

***Limitations and future research directions.*** While the study offers valuable insights, it is essential to acknowledge certain limitations. While the sample size of 813 respondents provides a substantial dataset for analysis, it is important to acknowledge that the sample was drawn from an online survey administered by a specialized company. This method can introduce selection bias, as respondents who are more comfortable with online surveys or have access to the Internet can be over-represented.

Another important limitation concerns the operationalization of “climate action” as a broad construct. Climate-related behaviors vary considerably in required effort, cost, and social acceptability, ranging from relatively simple actions such as recycling to more demanding behaviors such as reducing air travel or making dietary changes. As a result, individuals may express different levels of intention depending on the specific behavior considered, a pattern that an aggregated measure does not fully capture. Future research should therefore examine more specific categories of climate actions to better understand how boredom and other psychological factors influence distinct types of behavior.

In addition, the study relies on self-reported intention to act rather than actual behavior. Although intention is a well-established predictor of behavior, prior research highlights an intention-behavior gap, whereby individuals do not always translate intentions into concrete actions. Consequently, the findings should be interpreted with caution, and future studies are encouraged to incorporate behavioral measures or longitudinal designs to assess whether reduced boredom or increased engagement leads to actual climate-related actions over time.Next, the hypothesized relationships between climate change worry, environmental identity, and climate change boredom were not confirmed, suggesting that the interplay between these variables may be more complex than initially assumed. This underscores the need for further research to explore the nuanced connections between emotions, beliefs, and boredom in the context of climate change. Additionally, the findings revealed that a sense of climate-focused community was positively associated with climate change boredom, contrary to the hypothesized negative relationship. This unexpected result underscores the importance of investigating the specific communication strategies employed by climate-focused communities and how these strategies may affect individuals’ engagement and boredom. Further research is needed to understand the dynamics of community-based climate change messaging.

## 6. Conclusion

Climate change boredom significantly predicts individuals’ intention to take action, highlighting it as a key psychological barrier to engagement and underscoring the need to explicitly address and mitigate it. Building on this central finding, the study offers a clearer understanding of the complex interplay of psychological factors in the context of climate change engagement. The study casts light on a set of variables that predict climate change boredom: increased environmental identity, stronger beliefs in climate change, higher commitment to the goal of reducing it, lower sense of climate change-focused community, stronger beliefs in the self-efficacy of cooperation, perceived higher impact of climate change on health, younger age, and urban living environment.

Furthermore, the study highlights the importance of fostering environmental identities and the potential role of climate change worry as motivators of climate action, when appropriately framed. In line with prior research, worry may contribute to engagement, particularly when individuals feel capable of taking effective action and are provided with clear behavioral pathways; however, excessive worry or lack of guidance may be counterproductive and lead to disengagement. Understanding the dynamics between these variables can inform more effective strategies for engaging individuals in climate change mitigation efforts. Addressing climate change boredom enhances individual engagement and ensures long-term environmental security. A disengaged public weakens collective preparedness and policy implementation, making societies more vulnerable to climate-induced crises. Therefore, proactive strategies to maintain motivation and public interest while strengthening efficacy and actionable knowledge are needed to reinforce climate resilience and safeguard environmental security at the local and global levels. In particular, policymakers should move beyond one-size-fits-all approaches and adopt targeted communication and engagement strategies that reflect demographic differences (e.g., age, living environment) and varying levels of engagement, using tailored messages, locally relevant examples, and participatory formats to sustain public involvement in climate action. At the same time, interventions should aim not only to increase concern but also to strengthen efficacy beliefs, clarify goals, and establish actionable pathways, ensuring that individuals remain both motivated and capable of contributing to climate solutions.

## Supporting information

S1 Table Variables used in the questionnaire and the scores obtained from the survey. (DOCX)
