# Supplementary material for: Climate change boredom: Exploring its predictors and the psychological factors that influence intention to act
Source: PLoS One. 2026 May 27;21(5):e0348574. doi: 10.1371/journal.pone.0348574 (PMC13215543; doi:10.1371/journal.pone.0348574)
Supplement: S1 Table — (DOCX) [file pone.0348574.s001.docx]

**S1 Table. Variables used in the questionnaire and the scores obtained from the survey**

| Investigated variable | Questions in the questionnaire | Average score | Answer option |
| --- | --- | --- | --- |
| Environmental identity (IV1) | 14-item environmental identity scale, according to Clayton et al. [37] |  | 1= It is totally false in my case…… 7= It is totally true in my case |
|  | 1. I like to spend time outdoors in natural settings (such as woods, mountains, rivers, fields, local parks, lake or beach, or a leafy yard or garden). | 6.4 |  |
|  | 2. I think of myself as a part of nature, not separate from it. | 5.9 |  |
|  | 3. If I had enough resources such as time or money, I would spend some of them to protect the natural environment. | 5.9 |  |
|  | 4. When I am upset or stressed, I can feel better by spending some time outdoors surrounded by nature. | 6.2 |  |
|  | 5. I feel that I have a lot in common with wild animals. | 4.6 |  |
|  | 6. Behaving responsibly toward nature — living a sustainable lifestyle —is important to who I am. | 6.0 |  |
|  | 7. Learning about the natural world should be part of everyone’s upbringing. | 6.4 |  |
|  | 8. If I could choose, I would prefer to live where I can have a view of the natural environment, such as trees or fields. | 6.3 |  |
|  | 9. An important part of my life would be missing if I was not able to get outside and enjoy nature from time to time. | 6.3 |  |
|  | 10. I think elements of the natural world are more beautiful than any work of art. | 6.3 |  |
|  | 11. I feel refreshed when I spend time in nature. | 6.4 |  |
|  | 12. I consider myself a steward of our natural resources. | 5.8 |  |
|  | 13. I feel comfortable out in nature. | 6.3 |  |
|  | 14. I enjoy encountering elements of nature, like trees or grass, even when I am in a city setting. | 6.4 |  |
| Climate change beliefs (IV2) | 12 items adapted after Heath and Gifford [61] explored three categories of beliefs (climate change existence, the human-made character of its causes, and its consequences)  *a) Beliefs about climate change existence:* |  | 1= Totally disagree, ……, 7= Totally agree |
|  | 1. Climate change is felt in Romania. | 6.2 |  |
|  | 2. We have already observed some signs of climate change in Romania. | 6.2 |  |
|  | 3. It seems to me that the temperatures are currently higher than they were in the past (in Romania). | 5.9 |  |
|  | 4. It seems to me that the temperatures, the precipitation, and the start and end dates of the seasons are different now compared to how they were in my childhood (in Romania). | 6.3 |  |
|  | 5. I am pretty sure that climate change is happening globally. | 6.3 |  |
|  | *b) Beliefs about climate change causes:* |  |  |
|  | 6. Climate change is primarily due to natural causes, not human activity. (Reversed) (#) | 3.8* |  |
|  | 7. The leading causes of global climate change are human activities. | 5.5 |  |
|  | 8. Global warming is only a natural temperature fluctuation and is not caused by human activity. (Reversed) | 4.1* |  |
|  | 9. I am sure human activities are to blame for climate change. | 5.5 |  |
|  | *c) Beliefs about climate change consequences:* |  |  |
|  | 10. Despite what most scientists say, I believe there will be positive consequences for the environment because of climate change. (Reversed) | 3.9* |  |
|  | 11. The consequences of climate change will damage the natural environment. | 5.9 |  |
|  | 12. Climate change will generate serious negative consequences. | 5.3 |  |
| Environmental worry (IV3) | 10-item climate change worry scale, according to Stewart et al. [99] |  | 1= It is totally false in my case…… 7= It is totally true in my case |
|  | 1. I worry about climate change more than other people. | 5.3 |  |
|  | 2. Thoughts about climate change cause me to have worries about what the future may hold. | 5.3 |  |
|  | 3. I tend to seek out information about climate change in the media (e.g., TV, newspapers, internet). | 4.7 |  |
|  | 4. I tend to worry when I hear about climate change, even when the effects of climate change may be sometime away. | 5.2 |  |
|  | 5. I worry that outbreaks of severe weather may be the result of a changing climate. | 5.6 |  |
|  | 6. I worry about climate change so much that I feel paralyzed in being able to do anything about it. | 5.0 |  |
|  | 7. I worry that I might not be able to cope with climate change. | 4.8 |  |
|  | 8. I notice that I have been worrying about climate change. | 5.1 |  |
|  | 9. Once I begin to worry about climate change, I find it difficult to stop. | 4.4 |  |
|  | 10. I worry about how climate change may affect the people I care about. | 5.1 |  |
| Climate change goal commitment (IV4) | 9-item goal commitment scale according to Hollenbeck et al. [100], which was adapted to the climate change as follows: |  | 1= It is totally false in my case…… 7= It is totally true in my case |
|  | 1. It is difficult to consider the importance of stopping or reducing the effects of climate change. (Reversed) | 3.6* |  |
|  | 2. It is not realistic for me to expect to achieve the goal of stopping or reducing the effects of climate change. (Reversed) | 3.6* |  |
|  | 3. It is very likely that this goal of stopping or reducing the effects of climate change will be revised, depending on how things will go. (Reversed) (#) | 3.0* |  |
|  | 4. Frankly, I don't care if I don't achieve this goal of stopping or reducing the effects of climate change. (Reversed) | 5.0* |  |
|  | 5. I am strongly committed to pursuing this goal of stopping or reducing the effects of climate change. (#) | 5.1 |  |
|  | 6. It wouldn't take much to make me abandon this goal of stopping or reducing the effects of climate change. (Reversed) | 4.3* |  |
|  | 7. I think that the goal of stopping or reducing the effects of climate change is a good goal to shoot for. (#) | 5.6 |  |
|  | 8. I am willing to put forth a great deal of effort beyond what I’d normally do to achieve this goal of stopping or reducing the effects of climate change. (#) | 5.2 |  |
|  | 9. I don't have much to gain if I try to achieve this goal of stopping or reducing the effects of climate change. (Reversed) | 4.5* |  |
| Climate change self-efficacy of cooperation (IV6) | Four statements from Heath and Gifford [61] explored the self-efficacy of cooperation |  | 1= It is totally false in my case…… 7= It is totally true in my case |
|  | 1. There are simple things that I can do that will have a meaningful effect to alleviate the negative effects of global warming. (#) | 5.5 |  |
|  | 2. I believe that little things I can do will make a difference to alleviate the negative effects of global warming. (#) | 5.4 |  |
|  | 3. Even if I try to do something about global warming, I doubt if it will make any difference. (Reversed) | 4.1* |  |
|  | 4. There is very little I can do to mitigate the negative effect of global warming. (Reversed) | 3.8* |  |
| Climate change boredom (IV5) | 9-items from Thompson and Barton’s [101] study were adapted to climate change focus, as follows: |  | 1= It is totally false in my case…… 7= It is totally true in my case |
|  | 1. Climate change threats (such as the disappearance of some species of animals, plants, global warming, and melting glaciers) have been exaggerated. | 3.6 |  |
|  | 2. It seems to me that most environmental specialists are pessimistic and somewhat paranoid about climate change. | 3.8 |  |
|  | 3. The problem of the depletion of natural resources due to climate change is less severe than many people make it out to be. | 3.7 |  |
|  | 4. I find it hard to get concerned about climate change. | 3.6 |  |
|  | 5. Humans are dependent on nature to survive. (Reversed) (#) | 1.7* |  |
|  | 6. Most problems caused by climate change will solve themselves given enough time. | 3.6 |  |
|  | 7. I don't care about climate change. | 2.6 |  |
|  | 8. I’m opposed to programs to combat climate change (such as those related to environmental conservation, pollution reduction, and resource conservation). | 2.7 |  |
|  | 9. Too much emphasis has been placed on fighting climate change. | 3.1 |  |
| Intention to act against climate change (DV) | Four items from din Heath and Gifford [61] were used to investigate the general intention to act against climate change. To these, we added a set of six items for specific intentions to act: |  | 1= It is totally false in my case…… 7= It is totally true in my case |
|  | 1. I plan to take some actions to stop global warming. | 5.2 |  |
|  | 2. I personally do not intend to do much to stop global warming. (Reversed) | 5.0* |  |
|  | 3. I will make some efforts to mitigate the negative effects of global warming. | 5.4 |  |
|  | 4. I intend to take concrete steps to do something to mitigate the negative effects of global warming. | 5.3 |  |
|  | 5. I intend to eat less meat. (#) | 4.6 |  |
|  | 6. I intend to consume fewer products and services, in general (clothing, food, electronics, etc.). (#) | 4.8 |  |
|  | 7. I intend to reuse and recycle more. | 5.9 |  |
|  | 8. I intend to use carsharing (several people use the same car for common destinations) and travel less by car and more by train, bus, or bicycle. | 5.0 |  |
|  | 9. I intend to reduce my conventional energy consumption (for example, turn off the lights when I am not using them, buy electric cars, install photovoltaic panels, use less air conditioning in summer and less heat in winter). | 5.7 |  |
|  | 10. I intend to discuss the changes I am making to fight climate change. | 5.2 |  |
| Sense of climate-focused community (IV7) | Nine items were adapted after Coissard et al. [82]: |  | 1= It is totally false in my case…… 7= It is totally true in my case |
|  | 1. I consider myself to be a member of the department I work in. | 5.2 |  |
|  | 2. The department I work in is particularly motivating for me to be interested in climate change. | 4.0 |  |
|  | 3. I am happy to work in the department I work in. | 5.0 |  |
|  | 4. I consider that my department is one of the best departments in the company. | 4.7 |  |
|  | 5. People in my department are concerned with climate change. | 4.1 |  |
|  | 6. I consider myself part of a department for which climate change is a priority. | 4.0 |  |
|  | 7. I am very attached to the team of people who I work with regularly. | 4.9 |  |
|  | 8. I am very attached to the department as a whole | 4.8 |  |
|  | 9. I am very attached to the company in general. | 4.8 |  |
| Climate change influence on health (IV8) | 1. How much do you think climate change has affected your health (for better or worse)? (Reversed)  2. Do you think your health will deteriorate in the future due to climate change? | 4.7*  4.5 | 1. 1= It has deteriorated a lot, …, 7= It has improved a lot  2. 1= Definitely not, ………………….., 7= Definitely yes |
| Demographics (IV11,12,13) | Age (mean; SD) | 45.6; 13.3 | Open answer |
|  | Gender (percentage of total sample) | F: 51.8  M: 48.2 | F; M; Other/Prefer not to say |
|  | Living environment (percentage of total sample) | U: 88.7  R: 11.3 | Urban; Rural |
| IV: independent variable; DV: dependent variable; *: these average scores are calculated with reversed values of participants’ answers; #: items included in the questionnaire, but deleted from the PLS-SEM analysis, following the results of factor analysis (presented in section 4.2.) | | | |
